# The Helping Networks of Transgender Women Living with HIV

**DOI:** 10.1007/s10900-022-01179-0

**Published:** 2023-01-20

**Authors:** Miranda Hill, Jae Sevelius, Athena D. F. Sherman, Monique Balthazar, Meredith Klepper, Asa Radix, Greg Rebchook, Nathan Hansen

**Affiliations:** 1 Department of Medicine, University of California San Francisco, 550 16th St., San Francisco, CA 94158, USA; 2 Nell Hodgson Woodruff School of Nursing, Emory University, Atlanta, GA, USA; 3 Byrdine F. Lewis College of Nursing and Health Professions, Georgia State University, Atlanta, GA, USA; 4 John Hopkins University School of Nursing, John Hopkins University, Baltimore, MD, USA; 5 Callen-Lorde Community Health Center, New York, NY, USA; 6 Department of Health Promotion & Behavior, University of Georgia, Athens, GA, USA

**Keywords:** Community health interventions, Natural helping networks, Transgender women, HIV/AIDS, Social network analysis

## Abstract

Transgender women living with HIV face significant barriers to healthcare that may be best addressed through community-centered interventions holistically focused on their HIV-related, gender-related, and other important needs. Community health ambassador (CHA) interventions (education and training programs designed to engage communities and community leaders in health promotion) may be an effective option, though information about the natural helping networks of this vulnerable population is too limited to inform the implementation of this approach. This study uses social network analysis to describe the natural helping networks of transgender women living with HIV, their help-seeking patterns for HIV-related, gender-related, and ancillary resources, and the characteristics of potential network ambassadors. From February to August 2019, transgender women living with HIV in the US (N = 231) participated a 30-min online survey asking them to describe their natural helping networks (N = 1054). On average, participants were embedded within natural helping networks consisting of 4–5 people. They were more likely to seek help from informal network members vs. formal service providers (p < .01), and from chosen family and partners/spouses (p < .05) above other social connections. Older network members (p < .01), other transgender women (p < .05), and those with whom they regularly engaged face-to-face (p < .01) (vs. social technology) were identified as potential network ambassadors for HIV-, gender-related, and other important issues. These findings suggest an opportunity to develop CHA interventions that leverage existing help networks and potential network ambassadors to promote equitable access to HIV, gender-affirming, and other crucial resources among this medically underserved group.

## Introduction

Transgender women (people who are assigned male sex at birth who do not identify as male) experience substantial HIV inequities. One recent federal surveillance project across U.S. cities found that 40% of transgender women were living with HIV [1]. Multiple studies also report poorer clinical outcomes among transgender women living with HIV [2]. Generally, data suggest that access to clinical resources among transgender women living with HIV may be limited by (a) their knowledge of, and ability to access, a particular resource from potential helpers; (b) the scarcity of competent and gender-affirming resources available [3–5]; and (c) historical and anticipated experiences of intersectional stigma (e.g., HIV and/or gender-based stigma) [6, 7]. Moreover, social, and economic inequities among transgender women living with HIV translate into disparate needs for ancillary and support services that present additional barriers to HIV treatment. In one national study, researchers found that over 40% of transgender women living with HIV needed housing, transportation, and meal services [8]. The persistence of complex barriers to HIV, gender-affirming, and ancillary resources suggests a need for culturally responsive and community-centered interventions that increase access to care resources among transgender women living with HIV and their social networks.

Community health ambassador (CHA) interventions, which leverage indigenous helping networks to distribute resources within communities, are a promising intervention model for lowering barriers to equitable care and resource access among transgender women living with HIV [9, 10]. CHA intervention models have been widely implemented and successful in promoting diabetes prevention, tobacco cessation, and several other high-priority health issues among medically underserved groups [9–11]. Natural helpers, who typically are a well-known source of information and advice within the community of interest are recruited, educated, and trained to disseminate health information and resources throughout community networks. To our knowledge, there are no existing CHA programs within the literature that holistically focus on meeting the interconnected HIV-, gender-related, and other important needs of transgender women living with HIV, and there are limited descriptive data on their natural helping networks and help-seeking patterns to inform intervention development.

To address the above knowledge gaps and inform CHA intervention development, the present study seeks to characterize the natural helping networks and help-seeking patterns of US transgender women living with HIV in a national cross-sectional study. Social network research methods have been widely used for characterizing help-seeking and are well-suited for informing CHA intervention development due to the ability to map out details on structural and behavioral aspects of networks which determine the potential feasibility and success of this approach. When calculated, structural measures such as network size (defined by the number of network members) and density (defined by who knows each other within a network) are critical to understanding the extent to which a population can engage others for help (size) and how well-integrated they are within a community from which they can activate support from (density) [12]. Thus, network size and density are also useful indices for assessing (a) the viability of a community-based intervention vs. one that focuses squarely on individuals; and (b) the potential openness of a system to novel information, health promotion messages, and interventions [12]. Network methods also enable the exploration of complex help-seeking patterns. For example, research on psychiatric patients has found that people selectively activate support from different groups of people for health-related vs. non-health-related matters. Further, it has been found that people consult a select group of people for help with multiple issues [13]. Examining the characteristics of the people from whom transgender women living with HIV regularly go for HIV-, gender-, and other important issues expa nds our understanding of potential network ambassadors who could be educated and trained to disseminate multiple resources. Therefore, our aims are to describe (1) the structure and characteristics of three different social networks among transgender women living with HIV, consisting of the people from whom they regularly engage in discussions about HIV-related matters, gender-related matters, and other generally important (ancillary) matters (2) the characteristics of network members who are regularly engaged in discussing HIV-, gender-related, and important matters (3) the methods used to engage network members in discussions (in-person, online, etc.), (4) and the characteristics of natural helpers who are consulted for multiple issues (HIV-, gender-related, and/or other important matters).

### Participants

Participants were recruited between January and August 2019 for an online cross-sectional survey on HIV and relationships. Purposive sampling of transgender women who were living with HIV in the U.S. took place online and in person. Online recruitment involved sending out a description of the study and flyers to websites (Transgender Heaven), community-based organizations, and online social groups on social media sites consisting of transgender women and people living with HIV. In-person recruitment involved flyer and poster distribution to community-based organizations and at public community-led events that focused on transgender women in Atlanta, GA. Study materials directed potential participants to access the survey on Qualtrics via a provided link. Potential participants filled out an online consent form describing the study’s purpose, inclusion criteria, risks, and benefits that were on the first page of the survey. Those who consented to participate were automatically taken to an online eligibility screening survey. Eligible participants self-reported residing in the US, being at least 18 years of age or older, being diagnosed with HIV and prescribed antiretroviral therapy, identifying as women or transgender women, and being assigned male sex at birth.

Participants were administered a four-part survey which began by eliciting three different networks. Specifically, they were asked to list the initials of up to twenty-one people whom they regularly confided in about important (ancillary), then HIV, and finally, gender-related matters. The questions within the next section asked them to describe the people within their networks in terms of their race, gender, relationship role (e.g., family of origin, partner), and primary communication methods among other demographic characteristics. The following section asked them to select the people who knew each other across all networks. Participants were asked to provide personal demographic information at the end of the survey Participants received $20 to compensate for their time and participation. The University of Georgia’s Institutional Review Board approved all study procedures.

## Data Analysis

### Network Size

The size of each network (HIV-related, gender-related, and important matters) and a combined network (consisting of non-redundant people) were constructed from the number of people listed in response to the above questions. The maximum value for network size in this study was twenty-one (unique people within their combined network), with the lowest possible size being zero.

### Network Density

We calculated density (a continuous measure, ranging from zero to one, with zero indicating that no one person in a network is connected to anyone else, and a value of “one” indicating that all people with a given network are connected.) for each network and a combined network (consisting of all people listed) in Excel.

### Characteristics of Network Members and Communication Methods

Counts, proportions, and mean statistics for network composition were calculated in E-Net [14]. Participants identified several combinations of overlapping relationship roles within their networks (for example, a friend and co-worker). Thus, the primary relationship that the participant identified was used as the primary descriptor for network members with overlapping roles. Frequencies, means, and standard deviations of measures used to describe the sample and participant networks were calculated in the Statistical Package for Social Sciences v26 (SPSS) [15]. Next, descriptive statistics were calculated to determine the relative proportions of different types of people within the networks, network composition, structure, and relationship characteristics for each network.

### Potential Network Ambassador Characteristics

Potential network ambassadors were defined as network members who were regularly engaged from a combination of HIV-, gender, and/or other important matters. We conducted bivariate analyses (*X*^2^ and t-tests) in SPSS to compare and contrast the characteristics of network members who were “potential network ambassadors” versus those who were engaged for singular matters (network members).

## Results

### Sample Demographics

On average, participants (N = 231) were aged thirty-two years (SD = 7.58) and had been diagnosed with HIV for about four and a half years (SD = 5.9). Most identified their race as White (62%); nearly 30% identified as Black/African American and 9% as Latinx/a/o. Most were employed (67.8%) and stably housed (70.9%). Table 1 further details the sample demographics.

### Natural Helping Network Characteristics

Participants listed between 0 to 21 people whom they regularly engaged for HIV-related, gender-related, and important matters. On average, they discussed topics specific to HIV and gender with a mean of two to three people. Among these networks, the “important matters” network had the highest density (.62), followed by the “HIV network” (.48). The “gender” network was the least dense out of the three networks (.39). The participants’ combined networks had about five people in them and were moderately dense (.51), on average. There were no notable contrasts between the characteristics of those from whom participants sought help for any given issue. Most of the network members in each network and the combined network were White (44–45%) or Black/African American (42–45%) and about half of all people listed were also living with HIV (47–54%). A quarter to a third of helpers in each network were transgender women (26–31%); yet cisgender women comprised the highest proportion of helpers in the combined network and all other networks except for the gender specific network. The demographics characteristics of natural helping networks are further detailed in Table 2.

### Help-Seeking Patterns and Potential Network Ambassadors

Table 3 compares the characteristics of network members (defined as network members) who were regularly engaged for only one matter (HIV-, gender-related, or generally important matters) vs. those who were regularly engaged for multiple matters (defined as potential network ambassadors). On average, potential network ambassadors were older (*F* = 21.38, *p* = .002, [− 3.47, − .786]) (Table 3). There were also notable and significant gender differences between network members vs. potential network ambassadors (*X*^2^ = 11.46, *p* = .02). Transgender women and cisgender men were more likely to be potential network ambassadors, while cisgender women were less likely to be ambassadors, despite representing the largest proportion of genders within help-seeking networks. Transgender men were also less likely to be potential network ambassadors while representing a lower proportion of people who were listed within networks (Table 3). Chosen family (*X*^2^ = 33.26, *p* = .000) and partners/spouses (*X*^2^ = 6.17, *p* = .000) were more likely to be potential network ambassadors. Health providers/case workers (*X*^2^ = 11.58, *p* = .001), neighbors (*X*^2^ = 9.98, *p* = .002), and classmates (*X*^2^ = 26.15, *p* = .000) were less likely to be potential ambassadors. Potential network ambassadors were also more likely to be people whom participants knew for more than 2 years (*X*^2^ = 56.73, *p* = .000), felt very close to (*X*^2^ = 136.80, *p* = .000), and engaged through daily contact (*X*^2^ = 94.40, *p* = .000). Participants were most likely to engage potential network ambassadors via face-to-face interactions (*X*^2^ = 89.81, *p* = .000) and less likely to do so through social technology (e.g., social media, text messaging). Additional details on relationship and communication characteristics are outlined in Table 3.

## Discussion

### Natural Helping Network Characteristics

Our findings indicating that this sample of transgender women who are living with HIV were embedded in sizeable natural helping networks, which contrasts with past qualitative research indicating a potential for social isolation when seeking help among transgender women living with HIV due to HIV stigma [4].The authors of a study focused on HIV care engagement among transgender women stated that, “about half of the interview participants described profound isolation…when they were first diagnosed, often attributed to early family rejection and loss of community… [4]” Another recent study of help-seeking among transgender women found that while transgender violence survivors sought help from transgender and LGBQ families after experiences of violence, many of those who were living with HIV were reluctant to seek help from those resources due to former negative experiences with involuntary HIV disclosure, stigma, and social isolation within friendship and chosen family networks [16]. Nonetheless, our network analysis data highlight the possibility of developing intervention approaches to those that are network oriented vs. those that focus on individuals.

The size and density of the natural helping networks of transgender women living with HIV signify the necessary social infrastructure to support community health ambassador programs. As previously highlighted, measuring the degree to which people within a given network are connected to one another (density) is one structural index to describe how open a network might be to new information and interventions. It has been suggested elsewhere that highly dense networks diffuse intervention messages more easily due to the increased social connections between network members; however, highly dense networks may be less likely to adopt new information or interventions from external sources [12]. The density scores here suggest that at least half of the network members knew each other. This balance of interconnectivity and openness within the overall network (as indicated by the moderate density) is another promising, yet surprising finding—suggesting that the natural helping networks of transgender women living with HIV may be amenable to adopting CHA interventions while also having enough connections to diffuse intervention messages and resources. Nevertheless, structural quantitative measures should be interpreted with caution and in parallel with qualitative research. Qualitative research on HIV intervention strategies within House and Ball networks (LGBT kinship support networks centered around competitive community ballroom performances) have identified unique cultural factors that influence the adoption and uptake of evidence-based HIV prevention efforts [17]. Similar ethnographic and community engaged research is necessary to define the cultural context that will determine the successful uptake and implementation of CHA interventions for transgender women living with HIV [17, 18]. A sociocentric or “whole” network study may be a complementary approach to understanding the relationship dynamics with local communities that affect the success of a CHA intervention.

### Characteristics of Network Members and Communication Methods

The remaining findings describe the help-seeking communication methods and characteristics of natural helping network members. Similar to another network study with transgender women [19], we found a high prevalence of social technology use among transgender women living with HIV, whereby participants communicated with nearly 70% of their network via social technology (text messaging and social media). These communication channels present an opportunity to learn about the online support communities of transgender women living with HIV and signify the potential to promote equitable access to multiple resources through social technology driven interventions. However, our findings that highlight in-person contact as the preferred communication mode with potential network ambassadors suggest that a traditional in-person design may work best for harnessing the social influence of potential network ambassadors to promote messages that reach transgender women living with HIV. Additionally, our findings provide new information concerning the mixed HIV-serostatus within the natural helping networks of transgender women living with HIV (whereby nearly half of each network consisted of people who were living with HIV and people who were not living with HIV). This findings suggest the potential for status neutral approaches that integrate HIV prevention and treatment services, along with other assistance programs within a CHA intervention [20]. While HIV status neutral interventions are currently being implemented within clinical and organizational settings, our data highlight the potential for developing an approach that leverage existing helping networks to deliver those interventions. Furthermore, our analyses comparing the HIV serostatus of network members vs. potential ambassadors (in Table 3) suggest that people who are not living with HIV may have an equal likelihood of being potential network ambassadors.

### Characteristics of Potential Network Ambassadors

Future interventions may be improved by engaging natural ambassadors HIV, and/or gender-related and other important matters, such as partners and chosen family, in intervention development and treatment. More than two-thirds of network members (the majority of whom were chosen family and partners/spouses) were sought out for multiple types of help, suggesting that these key informal relations may be essential to driving survival, care engagement, and/or access to gender-related resources. Other studies have also highlighted the importance of chosen family in assisting transgender women with navigating various stressors such as financial strain, violence survivorship, and accessing HIV-focused and gender-affirming care [21] More research is needed to translate this growing evidence-base into CHA interventions that can be developed, tested, evaluated, and adapted to meet the needs of diverse underserved transgender communities.

## Limitations

Egocentric network data are commonly subject to bias due to the recall abilities of participants. The risk of misreporting network data may increase with the use of online self-report due to the absence of interviewer probing. Our findings are also limited to providing a cross-sectional snapshot of help-seeking and natural helping networks. Caution is warranted in interpreting data on social network structures and relationships given the volatility of these factors over time. The generalizability of our results are limited due to the convenience sampling strategy. Our examination of help-seeking was limited to discussing issues with network members whenever they came up. While engaging network members for discussing problem is a crucial primary step, further detail concerning the content and outcomes of those discussions would assist in understanding the receipt of resources. The use of an online data collection tool may also have facilitated access to the survey in some ways, but may have skewed participation to be more inclusive of people who were comfortable filling out an online survey. The recruitment of participants from community-based organizations may have also increased participation among people who are more connected to helping networks (vs. being socially isolated) via their connection to resources. Finally, asking participants who they regularly consult for “important matters” is a common and evidence-based question that is used to generate networks composed of meaningful helping relationships, how it is too vague to understand what type of important matters might be potential intervenable topics or resources.

## Conclusion

There is a need for interventions that broaden the reach of prevention and treatment strategies from medically underserved individuals to their communities. Data on the structure, help-seeking processes, and ambassadors within the natural helping networks of CHA intervention populations is integral to informing the development of interventions among transgender women living with HIV. The results found here suggest that US transgender women living with HIV are embedded within sizeable communities from which they seek out HIV, gender, and other important forms of help. This study also highlights the importance of key people and relationships, which may hold promise in developing interventions to improve the health of transgender women post-HIV diagnosis through community health initiatives. Importantly, our data show the potential for broadening the focus of future CHA interventions to also increase access to gender-affirming and ancillary resources within the natural helping systems of transgender women living with HIV.

## Figures and Tables

**Table 1:** Demographic characteristics of participants (N = 231)

Variable	Mean [Range]	SD
Age (years)	31.7 [21–59]	7.6
Time since diagnosis (years)	4.5 [0–35]	2.5
	N	(%)
*Data collection*		
Online	204	88.7
In-person	26	11.3
*Gender identity*		
Woman	16	7
Transgender woman	214	93
*Race*		
Black/African American	61	26.5
Latinx/a/o	21	9.1
White	143	62.2
Other (American Indian, Asian, Other)	5	2.2
*Annual household income*		
$0–10,000	41	17.8
$10,001–20,000	60	26.2
$20,000–30,000	54	23.6
> $30,000	74	32.2
*Work status*		
Unemployed	74	32.2
Employed	156	67.8
*Housing status*		
Homeless	20	8.7
Doubled-up	44	19.1
Stably housed	163	70.9
*Health insurance*		
No	61	26.5
Yes	168	73.0
*Relationship status*		
Never married	136	59.1
Widowed	2	0.9
Separated	7	3
Divorced	8	3.5
Living with a partner	36	15.7
Married	41	17.8

**Table 2:** Natural helping network characteristics (N = 1056)

Variable	Networks			
	Combined network	Important matters network	HIV matters network	Gender matters network
	Mean (SD)	Mean (SD)	Mean (SD)	Mean (SD)
Size	4.59 (4.64)	2.46 (1.99)	2.96 (2.56)	2.77 (2.43)
Density	0.51 (0.40)	0.62 (0.37)	0.48 (0.43)	0.29 (0.39)
Age (years)	35.19 (11.11)	35.81 (11.85)	36.11 (11.69)	34.87 (11.18)
	N (%)	N (%)	N (%)	N (%)
*Gender identity*				
Transgender woman	284 (27.3)	164 (29.2)	174 (25.8)	199 (31.4)
Transgender man	78 (7.5)	32 (5.7)	45 (6.7)	46 (7.3)
Gender non-conforming	32 (3.1)	20 (3.6)	16 (2.4)	13 (2.1)
Cisgender man	264 (25.4)	154 (27.5)	183 (27.2)	230 (36.3)
Cisgender woman	381 (36.7)	191 (34.0)	256 (38.0)	146 (23.0)
*Race*				
White	462 (44)	257 (45.7)	301 (44.4)	297 (46)
Black	436 (41.5)	239 (42.5)	302 (44.5)	272 (42.2)
Latinx/a/o	108 (10.3)	46 (8.2)	58 (8.6)	56 (8.7)
Asian/Pacific	28 (2.7)	11 (2.0)	11 (1.6)	11 (1.7)
Other	16 (1.5)	9 (1.6)	6 (0.9)	9 (1.4)
*Role*				
Origin family	99 (9.9)	69 (12.5)	68 (10.7)	57 (9.3)
Chosen family	116 (11.7)	83 (15.1)	84 (13.2)	77 (12.5)
Partner/spouse	57 (5.7)	44 (8.0)	39 (6.1)	46 (7.5)
Friend	385 (38.7)	233 (42.4)	252 (39.5)	239 (38.9)
Health provider	97 (9.7)	29 (5.3)	62 (9.7)	52 (8.5)
Co-worker	142 (13.4)	61 (10.8)	90 (13.2)	89 (13.7)
Neighbor	37 (3.7)	9 (1.6)	16 (2.5)	22 (3.6)
Classmate	51 (5.1)	19 (3.5)	21 (3.3)	24 (3.9)
Other	11 (1.1)	3 (0.5)	6 (0.9)	8 (1.3)
*HIV serostatus*				
Positive	475 (46.0)	256 (46.3)	304 (45.6)	340 (53.5)
Negative	558 (54)	297 (53.7)	362 (54.4)	296 (46.5)
*Communication*				
Email	46 (4.5)	18 (3.3)	25 (3.8)	22 (3.5)
In-Person	492 (4.4)	306 (55.7)	347 (53.4)	361 (57.7)
Phone	219 (21.6)	125 (22.8)	139 (21.4)	121 (19.3)
Social Media	128 (12.6)	51 (9.3)	67 (10.3)	62 (9.9)
Texting	119 (11.7)	46 (8.4)	64 (9.8)	59 (8.6)
Other	12 (1.2)	3 (0.5)	8 (1.2)	6 (1.0)

*Role* describes the primary relation-role selected by participants

**Table 3:** Characteristics of network members vs. ambassadors

Variables	Network member type		Chi^2^/*F*
	Network members^a^	Network ambassadors^b^	
	Mean (SD)	Mean (SD)	
Age (Years)	34.11 (10)	36.24 (11.9)	21.38***
	N (%)	N (%)	
*Gender identity*			
Transgender woman	127 (25.5)	156 (29.1)	11.46**
Transgender man	49 (9.8)^a^	28 (5.2)^b^	
Transgender non-conforming	19 (3.8)	13 (2.4)	
Cisgender man	118 (23.6)	145 (27.1)	
Cisgender woman	186 (37.3)	194 (36.2)	
*Race*			
White	215 (42.8)	244 (44.9)	45.9***
Black	178 (35.5)	258 (47.4)	
Latinx/a/o	74 (14.7)	33 (6.1)	
Other	35 (7)	9 (1.7)	
*Role*			
Origin family	39 (8.3)	59 (11.3)	2.61
Chosen family	26 (5.5)	90 (17.3)	33.26***
Partner/spouse	18 (3.8)	39 (7.2)	6.17**
Friend	170 (36.1)	212 (40.8)	2.28
Health provider/caseworker	62 (13.2)	35 (6.7)	11.58***
Co-worker	80 (15.8)	62 (11.4)	1.04**
Neighbor	27 (5.7)	10 (1.9)	9.978***
Classmate	42 (8.9)	9 (1.7)	26.15***
Other	7 (1.5)	4 (0.8)	–
*HIV serostatus*			
Positive	225 (45.5)	249 (46.6)	.143
Negative	270 (54.5)	285 (53.4)	
*Communication method*			
Email	34 (7)	11 (2.1)	14.09***
In-person	161 (33)	329 (62.8)	89.81***
Phone	107 (21.9)	112 (21.4)	.05
Social media	89 (18.2)	38 (7.3)	27.79***
Text-messaging	88 (18)	31 (5.9)	35.72***
Other	9 (1.8)	3 (0.6)	3.49
*Relationship length*			
≤ 6 months	55 (13.1)	33 (8.3)	56.73***
7–12 months	105 (25)	42 (10.5)	
1–2 years	89 (21.2)	61 (15.3)	
≥ 2 years	171 (40.7)	263 (65.9)	
*Contact frequency*			
≥ 1 year	39 (7.8)	14 (2.6)	94.40***
< once/year	23 (4.6)	9 (1.7)	
Bimonthly	70 (13.9)	35 (6.4)	
Monthly	94 (18.7)	49 (9.0)	
Weekly	160 (31.8)	183 (33.6)	
Daily	117 (23.3)	254 (46.7)	
*Tie strength*			
Not at all or not very close	102 (20.4)	26 (4.8)	136.80***
Somewhat close	113 (22.6)	59 (10.8)	
Close	128 (25.6)	108 (19.8)	
Very close	157 (31.4)	352 (64.6)	

* *p* < .10,

** *p* < .05,

*** *p* < .01

a Defined as network members engaged for only one matter (HIV-, gender-related, or generally important matters)

b Defined as network members engaged for multiple matters (HIV-, gender-related, and/or generally important matters)

## Data Availability

Data are available upon request from the first author.
